# Photosynthetic Performance and Vegetative Growth in a New Red Leaf Pear: Comparison of Scion Genotypes Using a Complex, Grafted-Plant System

**DOI:** 10.3389/fpls.2018.00404

**Published:** 2018-04-05

**Authors:** Francesca Tozzi, Ben M. van Hooijdonk, Donald S. Tustin, Luca Corelli Grappadelli, Brunella Morandi, Pasquale Losciale, Luigi Manfrini

**Affiliations:** ^1^Dipartimento di Scienze e Tecnologie Agro-Alimentari, University of Bologna, Bologna, Italy; ^2^The New Zealand Institute for Plant and Food Research Ltd., Hawkes's Bay, New Zealand; ^3^Consiglio per la Ricerca e l'Analisi dell'Economia Agraria, Centro di Ricerca Agricoltura e Ambiente, Bari, Italy

**Keywords:** red leaf, Asian and European pear, grafting system, gas exchange, plant vigor, dry matter partitioning

## Abstract

Leaf photosynthetic performance of a new red-skinned inter-specific hybrid pear variety called ‘PremP009’ (PIQA®BOO®) is presently unknown and therefore was compared to the Asian pear variety ‘Hosui’. The seasonal growth patterns and the final dry matter accumulation of all tree components were also investigated for both genotypes in their first year of growth after grafting. Leaf gas exchange and tree growth comparisons were assessed using an innovative grafted plant system, which involved a bi-axis tree with the presence of combinations of identical or mixed (one of each genotype) ‘PremP009’ and ‘Hosui’ scion genotypes grafted onto a single clonal rootstock (‘Buerre Hardy’ BA29). This experimental grafted plant system allowed a technique for comparing leaf photosynthesis of two scion genotypes on the same root system, thereby avoiding between-plant differences in plant water relations. ‘PremP009’ had higher leaf photosynthesis and higher leaf mass compared with ‘Hosui.’ However, by the end of the first year of growth, primary shoots of ‘PremP009’ were shorter with fewer nodes, corresponding to less dry weight gain in primary shoot leaves and stems. This vegetative behavior of ‘PremP009’ is likely a response to the smaller individual leaf area in the early season affecting light capture that greatly limits dry matter accumulation of young trees.

**HIGHLIGHTS**

- The bi-axis grafting technique never showed before in a scientific paper presents a strategic system for a comparative study of red/green leaf photosynthetic performance and related dry matter partitioning.

## Introduction

**Figure 1:**
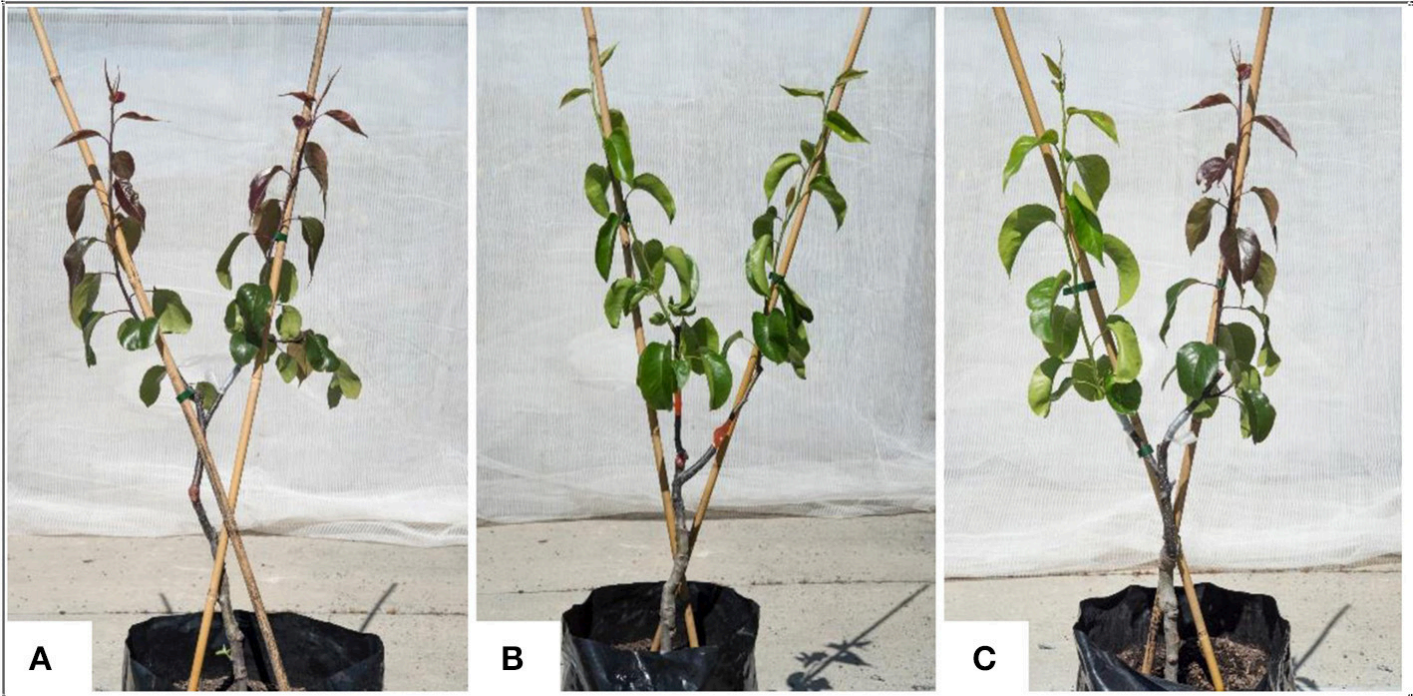
‘PremP009’ and ‘Hosui’ bi-axis trees grafted with either homogenous or mixed scion genotype (‘PremP009’ or ‘Hosui’) at shoot position (1 or 2). Figure **(A)** is a homogenous plant of ‘PremP009’ (H_P_) with red immature leaves. **(B)** Represents the homogenous plant of ‘Hosui’ (H_H_) with green leaves. **(C)** Represents a mixed tree (M) with ‘Hosui’ grafted at the lower nodal position (1) and ‘PremP009’ at the higher second nodal position (2). Trees are pictured 42 days after scion budbreak.

The new ‘PremP009,’ branded as PIQA®BOO®, is the first European (*Pyrus communis*) x Asian (*Pyrus pyrifolia*) interspecific pear (*P. communis x P. pyrifolia x P. bretschneideri)* released for cultivation. The intense red color of its skin is the main feature of ‘PremP009’ fruit, a quality trait appealing to many consumers. Young stems and juvenile leaves (Figure 1) exhibit red pigmentation as well, while mature leaves gradually show a darker green. Red leaves may result from an abundance of anthocyanins in the presence of relatively low chlorophyll levels, which may reduce overall leaf net carbon exchange rates thus limiting shoot growth, especially in early spring, i.e., when leaves are at their reddest (Martin et al., 1997).

Red-fruited cultivars have long been studied as their fruit color is perceived as advantageous to boost sales (Sugar, 1990; Feng et al., 2010). However, red-fruited pears have been reported to be more difficult to grow, less vigorous (Ing, 1987; Sugar, 1990; Zhang et al., 2012) and less productive, with lower yields per hectare than their green-fruited counterparts (Heitkamp, 1986; Burkhart and Willett, 1990; Martin et al., 1997). The lower vigor of red-fruited pear trees has been associated by several authors to reduced photosynthesis caused by the lower chlorophyll content (Willett, 1983; Rogers, 1985; Li et al., 2008). Martin et al. (1997) reported on the photosynthetic capacity of three red bud mutations and their three green parents. They found higher net photosynthesis and stomatal conductance, and higher Rubisco activity, for each green pear cultivar than their red-fruited bud sports. However, although differing in photosynthesis, often the growth of the three genetic pairs was not different (Martin et al., 1997). How red leaf genotypes may modify young tree growth and dry matter accumulation is still unknown. If red leafed genotypes have lower net carbon exchange rates, they should exhibit reduced growth rates (Marini, 1986). A preliminary and unpublished study of a red leaf seedling population grown on their own root systems suggested lower net photosynthesis for red than green leaf genotypes (Dr. Richard Volz, personal communication). Unfortunately, the stage of extension shoot growth at which leaf photosynthesis was measured for each genotype was unknown, and soil moisture content and stem water potential were not measured. Espley et al. (2012) on the other hand, found that transgenic red apple leaves (cv. Royal Gala) had higher photosynthesis than control plants. Through light response curves they showed higher electron transport rate for red leaves at increasing levels of photosynthetically active radiation (PAR). However, no data for plant water status and annual growth (dry mass) were presented.

Another factor that limits young tree dry matter accumulation is leaf area development, which may well differ between genotypes. In young apple trees, van Hooijdonk et al. (2015) reported that dry matter accumulation was limited by the rate of early-season leaf area development necessary for the interception of solar radiation for photosynthesis. Thus, measurement of node/leaf formation of the primary axes is as important as the measurement of leaf photosynthetic rates to understand and interpret the growth and physiological behavior of young trees. Source-sink relationships and regulation of carbon allocation determine growth and yield (DeJong and Grossman, 1995; Morandi et al., 2014b; Zhang et al., 2016). Carbon allocation is affected by the amount of available assimilate (source limitations) and by the organ's ability to utilize assimilates (sink limitation) (Patrick, 1988). Both these limitations respond to multiple internal (mainly genetic and physiological) and external (environmental) factors. Nowadays, all these variables are taken into account in models, developed for simulating the carbohydrates pathway. Thus, it is possible to estimate biomass accumulation to leaves, stem and root (Poorter et al., 2012), or the final fruit quality (i.e., Lescourret et al., 2011).

**Figure 2:**
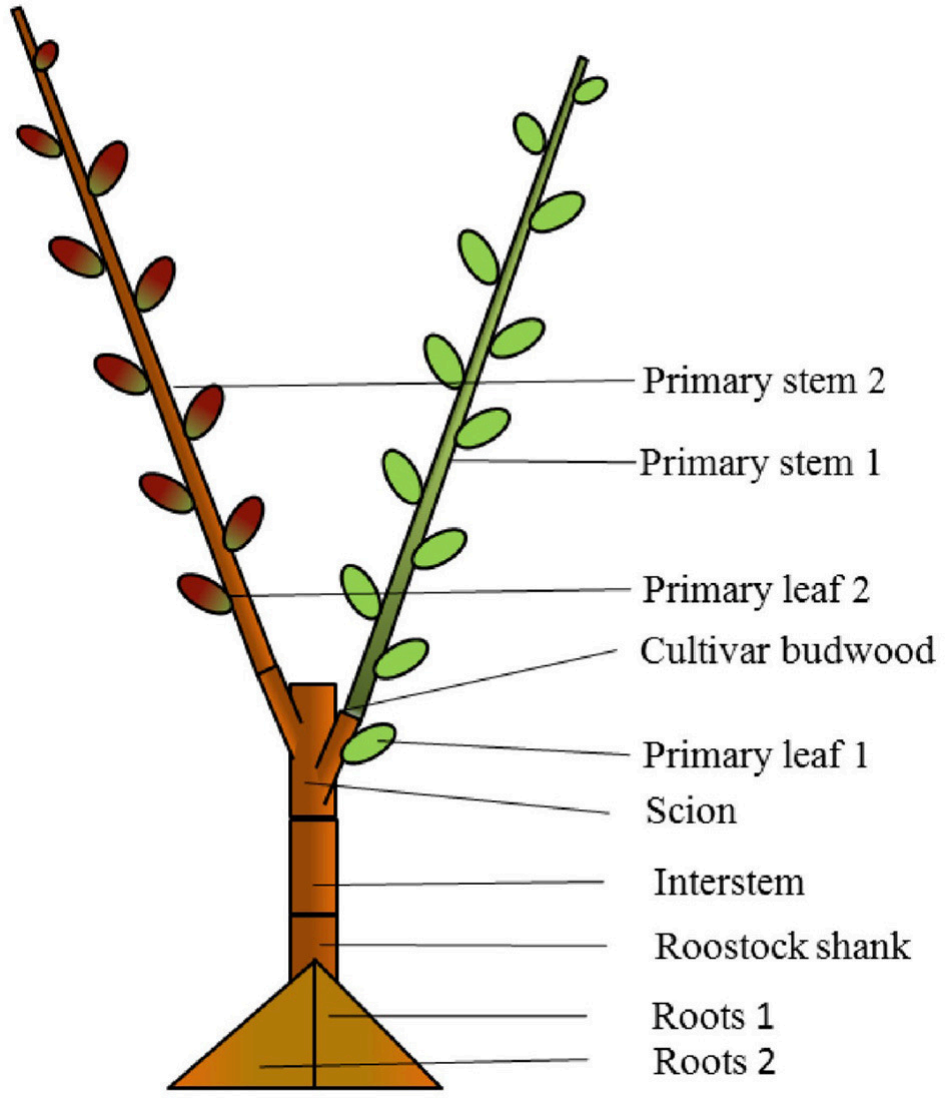
Composition of “mixed” bi-axis (M) tree with ‘Hosui’ grafted at the lower first nodal position and ‘PremP009’ grafted at the higher second nodal position. The figure represents how the tree has been cut for obtaining the final dry weight of individual plant's organs at growth cessation.

We hypothesize that red leaves of ‘PremP009’ have lower leaf photosynthetic performance limiting young tree vegetative development. The physiological behavior of ‘PremP009’ is unknown, and this study attempts to quantify the photosynthetic performance of its leaves, the relationship between photosynthesis and final dry matter accumulation/distribution considering the seasonal vegetative development during the first year of growth. The results are compared with the photosynthetic performance of ‘Hosui,’ a vigorous pear cultivar with typical green leaves. ‘PremP009’ and ‘Hosui’ share some physiological traits; they are easily managed in the orchard and they have a simple architecture of the tree; however, ‘Hosui’ leaves have never revealed the presence of the red pigments. Recently, there is increasing interest on split-canopy shapes, also known as bi-axis planting systems (Dorigoni et al., 2011), a similar tree shape has been adopted here. Multi-grafted trees developed with two main vegetative primary shoot axes were sampled for estimating whether the ‘PremP009’ leaf effectively has lower photosynthesis than ‘Hosui’ leaf, where all other within-tree factors are commonly regulated by the one rootstock/root system assuming equal plant, environmental and hydrological conditions. In pear, it has been pointed out that tree photosynthetic productivity can vary depending on the chosen rootstock (Losciale et al., 2008; Bosa et al., 2016). The multi-grafting system adopted here, is a new experimental technique for improving the physiological study of fruit trees and for elucidating at best any interaction between genotypes (Figure 2). To our knowledge, no previous study has used a similar multi-graft system for comparisons of photosynthesis and vegetative growth. Of added practical interest was whether the insertion of ‘Hosui,’ (hypothesized to have greater net photosynthesis and growth vigor), might contribute to overall total tree growth (dry mass) if grafted into a ‘PremP009’ tree.

## Materials and methods

### Plant material

The experiments were conducted during the 2014–2015 season at the Hawke's Bay Research Centre, The New Zealand Institute for Plant & Food Research Ltd., in Havelock North New Zealand (NZ). In late winter (mid-August 2012), scion genotypes of ‘PremP009’ were cleft grafted at 0.2 m height onto 1-year-old “Buerre Hardy” interstems on BA29 quince rootstocks. In August 2013, after 1 year in the nursery, ‘PremP009’ scions were cut back at 3–4 buds and grown as a bi-axis tree. During winter 2014, trees were lifted from the nursery and subsequently bench grafted using a cleft grafting machine (Raggett Industries, Gisborne, NZ) with two pear genotypes: ‘PremP009’ (*Pyrus communis x (Pyrus pyrifolia x Pyrus bretschneideri)*) and ‘Hosui’ (*Pyrus pyrifolia*) using the grafting combinations: “homogenous” (H) formed by grafting two identical scion genotypes per tree, or “mixed” (M) formed by grafting two different genotypes per tree. This determined three main treatments: H_P_ with ‘PremP009,’ H_H_ with ‘Hosui’ scion genotypes on both axes and M with one axis of each scion genotype. A further treatment subdivision related to shoot position, characterized by the numbers “1” and “2.” Position 1 is the primary axis originating closest to the root system and 2 the farthest (Figure 2) for both H and M plants. The subsequent treatments were: H_P_1, H_P_2, H_H_1, H_H_2, M_P_1, M_H_2, M_H_1 and M_P_2 generated from four different plant types: H_P_1_P_2, H_H_1_H_2, M_P_1_H_2, and M_H_1_P_2.

Grafted trees were planted into black polythene 50 l bags containing growing medium comprised: 0.8 composted bark, 0:2 pumice (by volume), 1 g l^−1^ 6-month slow release fertilizer, 3 g l^−1^ 9-month slow-release fertilizer (each 13 N, 5.7 P, 10.8 K, 1.2 Mg) (Nutricote r., Chisso-Assahi Fertilizer Co., Japan), and 0.6 g l^−1^ Magri-Trace (15 Mg, 3.2 Ca, 18 Fe, 3 Mn, 4 Zn, 3 Cu, 0.6 B, 0.08 Mo) (HortFert Plus Ltd, NZ) (van Hooijdonk et al., 2015).

### Primary axis growth curves and whole tree final dry matter accumulation

Diameter, length and node number of all primary axes were measured monthly throughout the growing season following the protocol described by van Hooijdonk et al. (2015) for the bi-axis nursery tree design. The diameters of the primary axes were measured with digital calipers and were used for calculating the circumference and the total shoot cross-sectional area (SCA) for each tree. The shoot length and node number measurements were taken from the shoot base, ending at the first unfurled leaf of the shoot apex beginning from October 21st, 2014 (28 DABB, days after bud break). The final growth assessment was measured on March 3rd, 2015 (early autumn), and subsequently the area of each leaf was measured using a leaf area meter (Li-3100, LI-COR Inc., USA). In addition, the node number of primary axes and small spurs were measured. ‘PremP009’ and ‘Hosui’ leaf discs (10 mm in diameter) were removed from mature (within the fifth and tenth nodal position) and young leaves (the upper three nodal positions), for observing possible differences due to the age effect in leaf disc dry weight (LDDW, g) and the specific leaf weight expressed as leaf mass area (LMA, g cm^−2^). Eighteen randomly selected trees per treatment were then sampled to obtain the total dry weight of root, rootstock shank, interstem scion, graftwood section, primary shoot leaves, primary shoot stem, small spurs, and sylleptic shoots from the primary stems (where present). Roots were cut in two parts, each part being that subtending its respective primary stem, for splitting the contribution of the two different scions to the growth of the roots. The dry weight for each scion genotype has been assigned to each part of the roots and primary shoot stem of the bi-axis trees, thus obtaining the weight of each of the two “halves” of the tree (Figure 2, **Tables 4, 5**). All samples were oven dried at 60°C to a constant weight.

### Gas exchange and water potential measurements

On January 19th (104 DABB) and February 15th (139 DABB) 2015, gas exchange measurements were performed on six leaves of each plant type (see experimental design). Leaves selected were healthy and fully-expanded, and were measured at similar nodal positions on each primary shoot (i.e., midpoint) to ensure a similar age and light exposure. Measurements were carried out during the day at 9:00 (from 9:00 to 10.00), 13:00 (from 13:00 to 14:00) and 16:00 (from 16.00 to 17:00). Cumulative photosynthesis (∑_A_) and transpiration (∑_E_) over the time of daily measurements (from 9:00 to 17:00) were also calculated. As described by Losciale et al. (2010) and Cano et al. (2014) for other photosynthesis related parameters, the formula used was:

(1) $$\begin{array}{r} \underset{y}{\sum }=\overset{t_{1}}{\underset{i=t_{0}}{\sum }}{\left(\frac{y_{t_{0\;}+}{\;y}_{t_{1}}}{2}\right)}_{i}+\overset{t_{2}}{\underset{i=t_{1}}{\sum }}{\left(\frac{y_{t_{1\;}+}\;y_{t_{2}}}{2}\right)}_{i} \end{array}$$

where *y* is the variable A or E, *t*_0_ corresponds to 9:30, *t*_1_ to 13:30 and *t*_2_ to 16:30.

Leaf gas exchange was measured with a portable photosynthesis system (Li-Cor 6400, Lincoln NE, USA), at a CO_2_ concentration similar to the environment (375 ppm), while radiation (PAR) was maintained constant at 1,000, 1,600, and 1,200 μmol m^−2^s^−1^ at 9:00, 13:00, and 16:00, respectively, representative of the light intensity at these times. During the gas exchange measurements, soil moisture was monitored with TDR sensor, (Time Domain Reflectometry) and quantification of the midday stem water potential (SWP) was performed by a pressure chamber (Soil Moisture Equipment corp., Santa Barbara, California, USA) on six leaves of each genotype according to the methodology described by McCutchan and Shackel (1992) and by Naor et al. (1995).

### Photosynthetic light response curves and A/C_i_ measurements

The photosynthetic light response curves were gathered on the 26th and the 27th of February 2015 and were made using a portable photosynthesis system (Li-Cor 6400, Lincoln NE, USA), at a CO_2_ concentration similar to the environment (375 ppm), following the protocol of Campbell et al. (1992). The sequence of the light intensity was 2,000, 1,500, 1,000, 700, 500, 300, 100, 50 μmol m^−2^s^−1^. A regression analysis was used to evaluate the response of leaf photosynthetic rate at different irradiances. Data were analyzed according to the model described by Corelli Grappadelli and Magnanini (1993) where:

$$\begin{array}{l} \text{A}=\text{B}0\left(\text{B}1-\text{exp}\left(-\text{B}2^{*}\text{PAR}\right)\right) \end{array}$$

and B0, B1, B2 represent estimated parameters. A/C_i_ response curves were completed on March 3rd, 2015 and the measurements were performed at varying CO_2_ concentrations (375, 200, 100, 60, 2,000, 1,800, 1,500, 1,200, 800, 500, 375 ppm) using a PAR at 1,600 μmol CO_2_ m^−2^s^−1^. The A/C_i_ data were used to estimate the maximum rate of RuBisCo (ribulose-1,5-bisphosphate carboxylase/oxygenase) carboxylation activity (V_max_), and the maximum rate of electron transport driving RuBisCo regeneration (J_max_) as described by Farquhar et al. (1980). For light response and A/C_i_ curves, data were obtained from measurements on both ‘PremP009’ and ‘Hosui’ between 10:00 and 13:00, on well exposed and healthy leaves located at similar nodal positions, corresponding approximately to the middle of each primary axis.

### Experimental design and data analysis

The experiment was a completely random design with six plant replicates per treatment. Three main treatments were: H_P_, H_H_ (6 plants each treatment) with ‘PremP009’ or ‘Hosui’ scion genotypes in both axes, and M (6 plants) (Figure 2). The effect of treatments on whole-tree growth, allometry, dry matter accumulation and gas exchange parameters was evaluated by a two-way ANOVA (*P* ≤ 0.05) followed by Student-Newman-Keuls for mean separation. Light responses, A/C_i_ curves and LMA determinations were undertaken only on M trees for testing genotype behavior with no rootstock interaction allowing respectively a regression analysis and a one-way ANOVA (*P* ≤ 0.05) followed by Student-Newman-Keuls for mean separation. A further analysis based on shoot position, characterized by the numbers “1” and “2,” where position 1 is the primary axis originating closest to the root system and 2 the farthest (Figure 2) was undertaken for both H and M treatments. The subsequent treatments were: H_P_1, H_P_2, H_H_1, H_H_2, M_P_1, M_H_2, M_H_1, and M_P_2 with 6 leaves or shoots analyzed for H plants and 3 for the M trees. In this last case, an unbalanced three-way ANOVA (*P* ≤ 0.05) was performed on the gas exchange measurements for the three measurement times each day and on the dry matter accumulation data. The factors analyzed were plant genotypes, plant types and shoot positions.

## Results

### Light and carbon response curves, gas exchange and stem water potential

**Figure 3:**
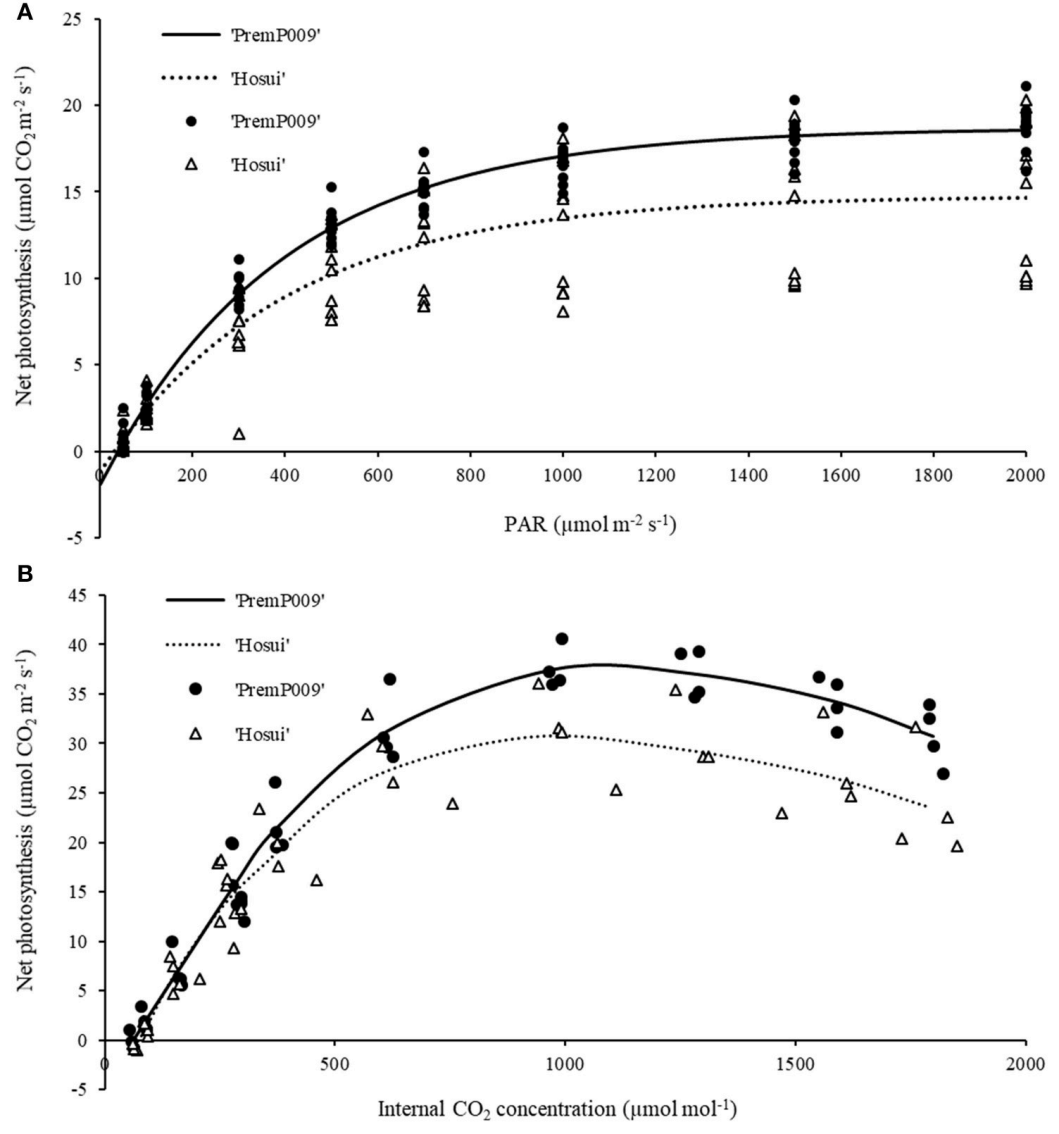
Light response curve **(A)** at ambient CO_2_ concentration (375 ppm) and carbon assimilation curve **(B)** at PAR 1,600 μmol m^−2^s^−1^. Lines represent the fitted model and symbols denote real data. Each curve represents the average of 4–6 leaf replicates of mixed (M) trees.

For the light and carbon response curves, only leaves within the M trees have been used, to observe the general photosynthetic response and to compare the photosynthetic efficiency of each genotype (‘PremP009’ and ‘Hosui’) on the same root system. Fitting the light response model to leaf photosynthetic rates at different irradiances resulted in an *R*^2^ of 0.98 for ‘PremP009’ and 0.75 for ‘Hosui,’ at ambient CO_2_ concentration (Figure 3A). Light saturation was recorded at circa 1,200 μmol m^−2^s^−1^ for both genotypes. The A/C_i_ curves (Figure 3B) revealed that J_max_ (Table 1) differed between genotypes, being higher for ‘PremP009’ leaves. There was no genotypic difference in the values of compensation point (CP) for light and CO_2_, which were 50 μmol m^−2^s^−1^ for PAR and around 60 ppm for CO_2_ (Figure 3) (Farquhar et al., 1980).

**Table 1:** Maximum rubisco carboxylation rate (V_max_) and maximum electron transport rate (J_max_) in ‘PremP009’ and ‘Hosui’ pear leaves at PAR 1,600 μmol m^−2^s^−1^.

**Genotype**	**V_max_**	**J_max_**
‘PremP009’	73.9	180.9
‘Hosui’	71.6	139.1
Significance	ns	^**^

*Differences for each genotype, values rapresent a mean of four replicates*.

** *P < 0.001; ns, not significant*.

For mature leaves of both LDDW and LMA, ‘PremP009’ showed higher values (28 mg and 35.6 mg cm^−2^, respectively) than ‘Hosui’ (21 mg and 26.9 mg cm^−2^). In contrast to mature leaves, young ‘PremP009’ leaves have lower LDDW and LMA than ‘Hosui’ leaves (Table 2).

**Table 2:** Leaf disc dry weight (g) and leaf disc mass area (g cm^−2^) of young and mature leaves of ‘PremP009’ and ‘Hosui’ pear.

	**Young leaves**		**Mature leaves**	
	**Leaf disc dry weight**	**Leaf disc mass area**	**Leaf disc dry weight**	**Leaf disc mass area**
**Genotype**	**(g)**	**(g cm^−2^)**	**(g)**	**(g cm^−2^)**
‘PremP009’	0.015	0.0191	0.028	0.0356
‘Hosui’	0.0161	0.0205	0.0211	0.0269
Significance	^*^	^*^	^*^	^*^

*Differences for each genotpe, values represent a mean of four replicates*.

* *P < 0.05*.

**Figure 4:**
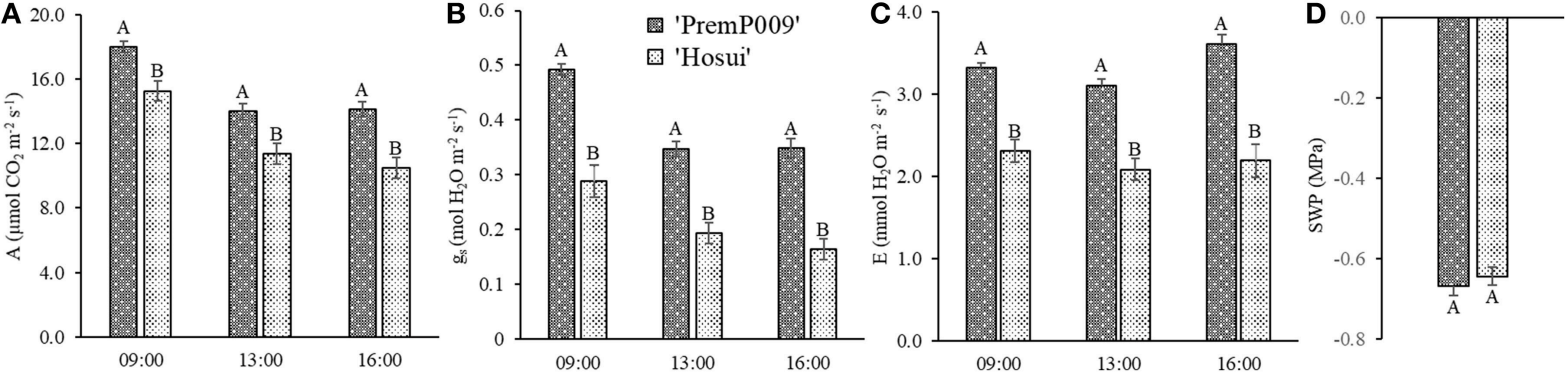
Mean (± Standard Error, SE) net photosynthesis (A) **(A)**, stomatal conductance (g_s_) **(B)**, transpiration (E) **(C)** at 9:00, 13:00, and 16:00 on 2015 January 15th, and midday stem water potential (SWP) **(D)** for the two genotypes. Each bar represents the mean ± SE of four replicates of the three plant types.

**Figure 5:**
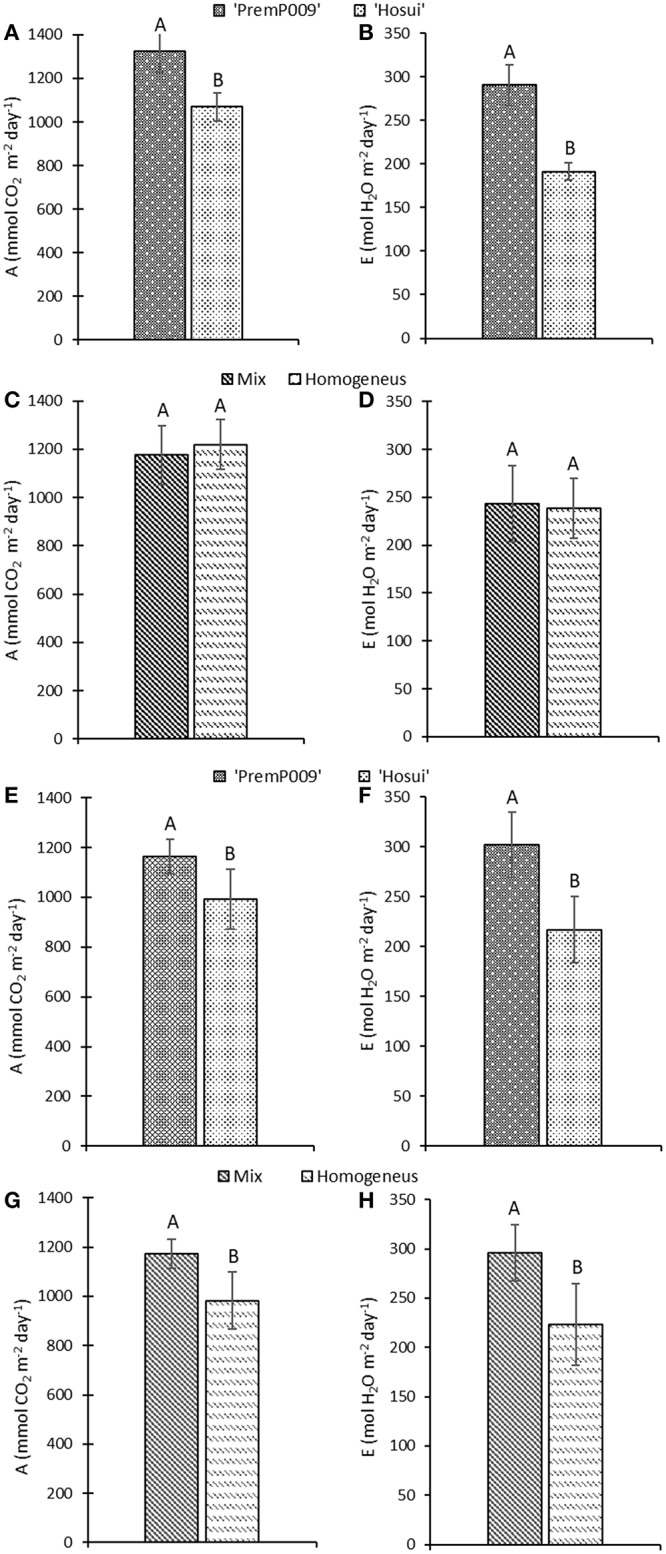
Cumulative net photosynthesis (A) and transpiration (E) (±Standard Error, SE) over the time of daily measurements on January 15th **(A–D)** and February 19th **(E–H)**. Measurements consider leaf genotype (‘PremP009’ and ‘Hosui’) and plant type (Homogeneous or Mixed). Each bar represents the cumulative mean ± SE of four replicates. Letters on the histograms indicate the mean separation. *P* < 0.05.

Photosynthesis of individual leaves was measured three times during the day in mid-January and late February. Data were analyzed by a three-way ANOVA of the factors leaf genotype, plant type, and shoot position. On January 15th, the measurements were significantly different only for leaf genotype, with no interactions among the other factors (Figure 4). Figure 4A shows that ‘PremP009’ overrated ‘Hosui’ at the 3 measured times. At 9:00, the highest net photosynthetic rate (A) of 18.0 μmol m^−2^s^−1^ was found for ‘PremP009,’ whereas ‘Hosui’ reached a peak at 15.2 μmol m^−2^s^−1^ (Figure 4A). Throughout the day for both genotypes, net photosynthesis declined, reaching lower rates. Net photosynthesis (A), stomatal conductance (g_s_) and transpiration (E), in fact, were greater for ‘PremP009,’ with the major peak at 0.49 mol m^−2^s^−1^ and 3.6 mmol H_2_O m^−2^s^−1^, respectively (Figures 4B,C). Despite differences in gas exchanges, no differences were found for SWP (stem water potential) between the plant genotypes (Figure 4D). If SWP is evaluated on the basis of the plant type factor (H_P_, H_H_, and M) over the 2 measurement dates, some differences appear. On January 15th, SWP did not differ between the three plant types, while on February 19th, H_P_ had a higher SWP of −0.58 MPa (Figure S1). On February 19th, the three-way ANOVA analysis indicated interactions between the factors as presented in Table 3. Taking into account the leaf genotype, ‘PremP009’ had higher A, g_s_, and E compared to ‘Hosui’; the only exception being at 9:00 h, when A and E were not different (Table 3). Plant type showed a predominant trend for M leaves to have higher A, g_s_, and E compared to H leaves. As shown in Table 3, these trends occurred almost all day long excluding A at 9:00, and independently from the genotype or shoot position. Comparing stomatal conductance, at 16:00 ‘PremP009’ in 1 and 2 position had higher values than comparable ‘Hosui’ positions. An interaction among plant type, leaf genotype and shoot position occurred for g_s_ at 16:00: ‘PremP009’ had greater average g_s_ than ‘Hosui,’ particularly when in the M trees, with the greatest value of 0.498 mol m^−2^s^−1^ on ‘PremP009’ leaves within M trees at position 2 (Table 3). A cumulative calculation of leaf photosynthesis and transpiration has been provided for a consolidated understanding of the gas exchange measurements in January 15th and February 19th. Each histogram represented in Figure 5 is the integration of net photosynthesis (Σ_A_) and transpiration (Σ_E_) of each leaf measured (Losciale et al., 2010). In the three-way ANOVA, no interactions were observed; leaf genotype in January and February and plant type in February showed differences (Figure 5). In general, ‘PremP009’ genotype recorded higher values of A and E than ‘Hosui’ genotype in both dates (Figures 5A,B,E,F). Regarding plant type, no differences were found in January (Figures 5C,D). In February (Figures 5G,H), leaves within the M trees had larger cumulated values for A and E than H ones.

**Table 3:** Net photosynthesis (A), stomatal conductance (gs) and transpiration (E) in February for the three factors: plant type, genotype and shoot position of bi-axis pear trees.

	**A (**μ**mol CO**_**2**_ **m**^**−2**^ **s**^**−1**^**)**										**g**_**s**_ **(mol H**_**2**_**O m**^**−2**^ **s**^**−1**^**)**										**E (mmol H**_**2**_**O m**^**−2**^**s**^**−1**^**)**									
			**Plant genotype**										**Plant genotype**										**Plant genotype**							
**Hour**	**Plant type**	**Shoot position**	**‘PremP009’**		**‘Hosui’**		**Plant type × shoot position**	**↓**	**Plant type mean**	**↓**	**Plant type**	**Shoot position**	**‘PremP009”**		**‘Hosui’**		**Plant type × shoot position**	**↓**	**Plant type mean**	**↓**	**Plant type**	**Shoot position**	**‘PremP009’**		**‘Hosui’**		**Plant type × shoot position**	**↓**	**Plant type mean**	**↓**
09:00	Mixed	1	14.3		12.7		13.47		13.93		Mixed	1	0.564		0.347		0.455		0.613	a	Mixed	1	4.00		2.67		3.34		3.86	a
		2	14.5		14.3		14.4					2	0.856		0.687		0.772					2	4.28		4.49		4.38			
		Mean →	14.4		13.5							Mean →	0.710		0.517							Mean →	4.14		3.58					
	Homogeneous	1	14.0		10.9		12.46		13.16		Homogeneous	1	0.376		0.24		0.308		0.374	b	Homogeneous	1	3.09		2.24		2.67		2.94	b
		2	14.5		13.3		13.86					2	0.591		0.291		0.441					2	3.83		2.59		3.21			
		Mean →	14.3		12.1							Mean →	0.483		0.265								Mean →	3.46		2.42				
	Shoot position	1	14.1		11.8		12.96				Shoot position	1	0.470		0.293		0.382	b			Shoot position	1	3.55		2.45		3.00			
	mean	2	14.5		13.8		14.31				mean	2	0.723		0.489		0.606	a			mean	2	4.05		3.54		3.79			
		General mean →	14.3		12.8							General mean →	0.597	a	0.391	b						General mean →	3.80		3.00					
13:00	Mixed	1	13.8		13.7		13.7		13.16	a	Mixed	1	0.518		0.410		0.464		0.443	a	Mixed	1	3.34		3.05		3.19		3.12	a
		2	13.6		11.6		12.6					2	0.610		0.235		0.423					2	3.77		2.31		3.04			
		Mean →	13.7		12.6							Mean →	0.564		0.322							Mean →	3.55		2.68					
	Homogeneous	1	11.5		7.2		9.3		10.14	b	Homogeneous	1	0.343		0.145		0.244		0.264	b	Homogeneous	1	2.79		1.48		2.13		2.29	b
		2	12.2		9.6		10.9					2	0.387		0.179		0.283					2	3.08		1.83		2.45			
		Mean →	11.9		8.4							Mean →	0.365		0.162							Mean →	2.93		1.65					
	Shoot position	1	12.6		10.4		11.54				Shoot position	1	0.430		0.277		0.354				Shoot position	1	3.06		2.26		2.66			
	mean	2	12.9		10.6		11.76				mean	2	0.498		0.207		0.353				mean	2	3.43		2.07		2.75			
		General mean →	12.8	a	10.5	b						General mean →	0.465	a	0.242	b						General mean →	3.24	a	2.17	b				
16:00	Mixed	1	12.2		12.9		12.52		12.96	a	Mixed	1	0.307	bc	0.400	ab	0.353		0.351	a	Mixed	1	3.47		2.55		3.48		3.35	a
		2	14.7		12.2		13.4					2	0.498	a	0.199	c	0.349					2	3.31		2.19		3.22			
		Mean →	13.4		12.5							Mean →	0.402		0.299							Mean →	3.67		3.03					
	Homogeneous	1	12.0		8.6		10.31		11.11	b	Homogeneous	1	0.311	bc	0.148	c	0.230		0.232	b	Homogeneous	1	3.27		2.34		2.53		2.58	b
		2	13.0		10.8		11.9					2	0.290	bc	0.178	c	0.234					2	3.23		2.16		2.64			
		Mean →	12.5		9.7							Mean →	0.300		0.163							Mean →	3.19		1.98					
	Shoot position	1	12.1		10.8		11.42				Shoot position	1	0.309	a	0.274	b	0.291				Shoot position	1	3.34		2.66		3.00			
	mean	2	13.8		11.5		12.65				mean	2	0.394	a	0.188	b	0.291				mean	2	3.51		2.35		2.93			
		General mean →	13.0	a	11.1	b						General mean →	0.351	a	0.231	b						General mean →	3.43	a	2.51	b				

*Arrows indicate the direction of the reading. Letters indicate differences for the three factors analyzed, and the interactions*.

*^a^Mean separation by the Student-Newman-Keuls test; P < 0.05*.

### Dry matter accumulation and distribution

**Figure 6:**
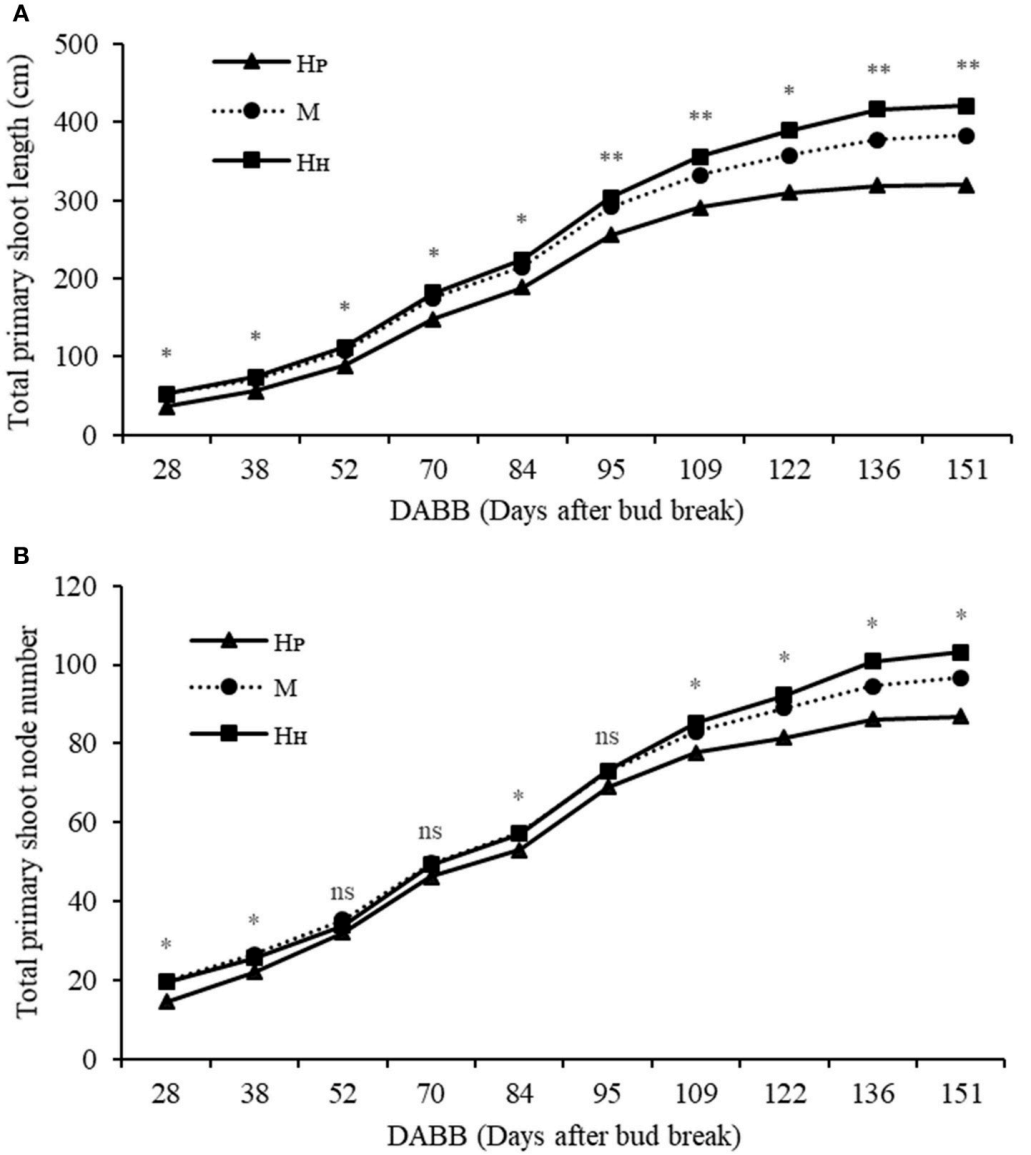
Total primary shoot length (cm) **(A)**, total node number **(B)** of ‘PremP009’ and ‘Hosui’ homogenous (H_P_ and H_H_) and mixed plant (M). Each point represents the average of 6 replicates. Asterisks indicate significant differences between treatments (^*^P < 0.05; ^**^P < 0.01). ns, not significant.

**Figure 7:**
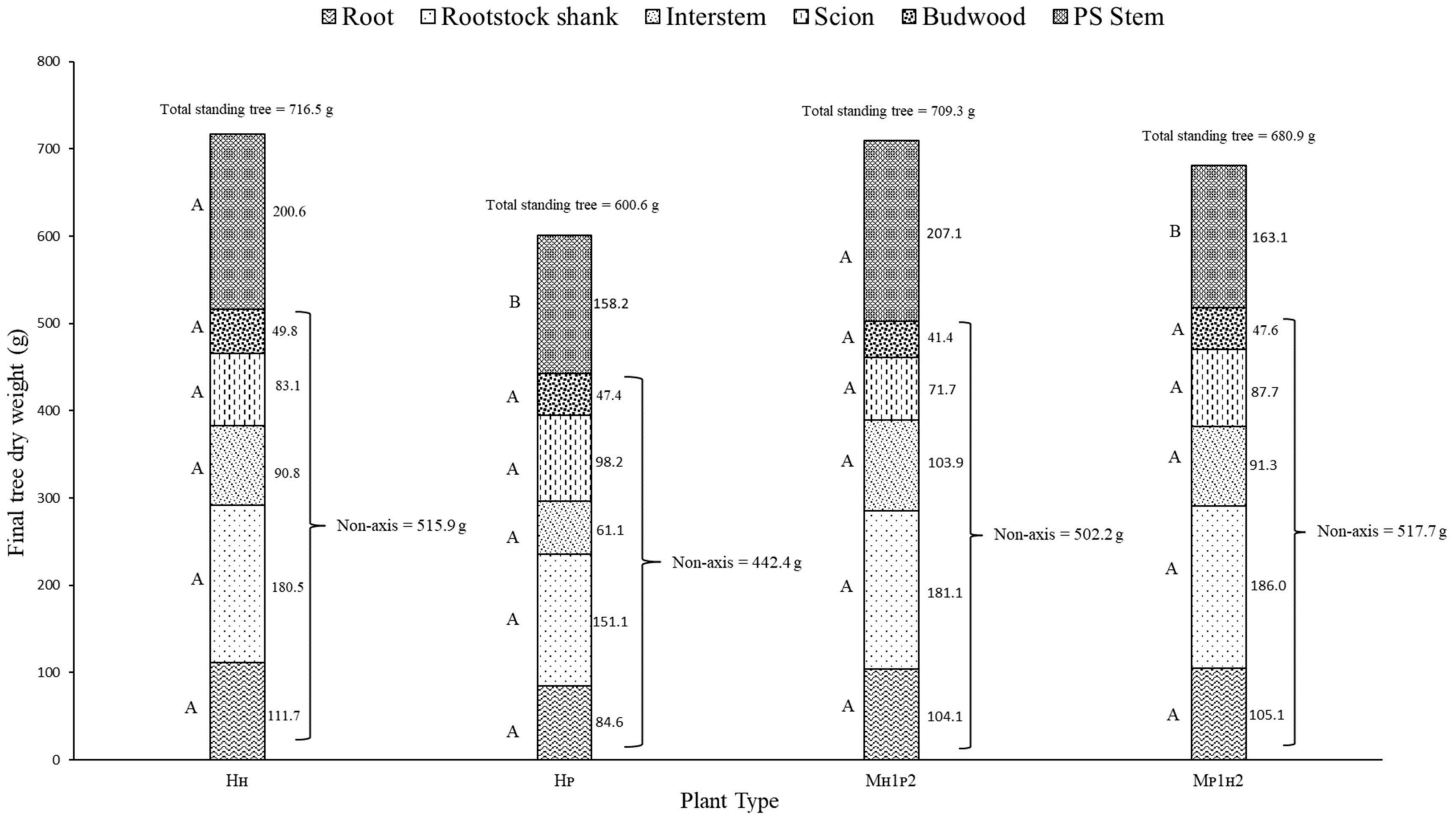
Total standing tree dry weight of ‘PremP009’ and ‘Hosui’ homogenous (H_P_ and H_H_) and the two combinations of M plant type (M_H1P2_ and M_P1H2_). Letters on the left side of the histograms indicate the mean separation between each part of the plant. *P* < 0.05.

Different components of plant growth and dry matter accumulation have been considered for the three plant types H_P_, H_H_, and M (see Figures 1, 2 for plant identification). Small sprout and sylleptic shoots (shoots that develop from a lateral bud without any period of dormancy) have not been considered part of the primary shoot axis and so have been excluded in the representation of scion extension growth. The curves presented in Figure 6 show the seasonal development of the mean total primary axis length and node number (the sum of the two axes) of H_P_, H_H_ and M plants describing the overall growth behavior of each plant type. Differences in primary axis growth among the treatments were measurable by 28 DABB with H_P_ shoots being shorter than H_H_ and M, with fewer nodes (Figures 6A,B). From 28 to 109 DABB, primary axis length and node number of H_H_ and M were similar but greater than H_P_ (Figures 6A,B). From 109 DABB until the end of the season, H_H_ developed the longest shoots, with the most nodes (Figures 6A,B). A regression analysis between final tree dry weight and total leaf area (Figure S2) showed a positive relationship. The 3 tree types occupy distinct positions within the graph: H_P_ trees in the lowest region, H_H_ with the highest values and M trees intermediate. In Figure 7, each histogram shows the final total standing tree dry weight as the sum of the non-axis parts (roots, rootstock shank, interstem and scion) plus the total primary stem dry weight of H_P_, H_H_ and the two combinations of M plant types (M_H_1_P_2 and M_P_1_H_2). Trees of H_H_ and M_H_1_P_2 had the greatest values of total primary stem dry weight of 200.6 and 207.1 g, while H_P_ and M_P_1_H_2 were 158.2 and 163.1g respectively. No difference between plant types has been found regarding the components of the non-axis part (Figure 7).

### Plant development and dry matter accumulation based on primary axis position

**Figure 8:**
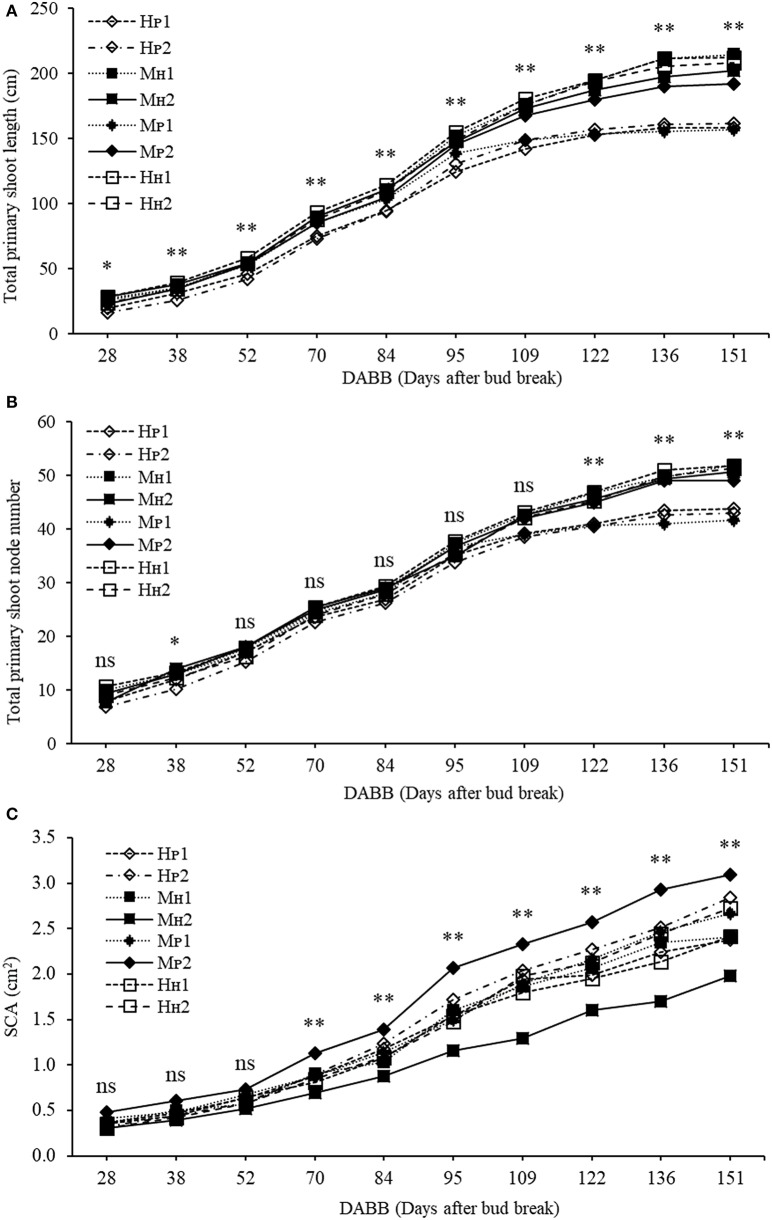
Total primary shoot length (cm) **(A)**, total node number **(B,C)** scion cross-sectional area (cm^2^) (SCA) of ‘PremP009’ and ‘Hosui’ considering genotype (‘PremP009’ and ‘Hosui’), type of grafting (homogenous and mixed) and shoot position (position 1 or 2). Each point represents the average of 6 replicates for homogenous and 3 for mixed trees. Asterisks indicate significant differences between treatments (^*^P < 0.05; ^**^P < 0.01). ns, not significant.

The three-way ANOVA of tree growth and dry matter accumulation revealed a positive 3-way interaction between tree type, genotype and shoot position (Table 4). Thus, an analysis of the scion extension growth in each tree combination identified by the primary shoot position has been undertaken (Figure 8 and Tables 4, 5). Small spurs and sylleptic shoots have not been considered as a part of the primary shoots and are excluded in the representation of scion extension growth. From 28 to 84 DABB, M_H_1, M_H_2, and H_H_1 developed the longest shoots, whereas H_P_2 the shortest (Figure 8A). From 95 DABB until the end of the season, H_H_1, H_H_2, M_H_1, M_H_2, and M_P_2 developed the longest shoots (Figure 8A). At 151 DABB, M_P_1, H_P_1, and H_P_2 had the shortest shoots. The mean node number per primary shoot was similar among all the treatments until 109 DABB (Figure 8B). At 38 DABB only, H_P_2 had fewer nodes than M_P_2 and H_H_1. From 122 DABB, H_P_1, H_P_2, and M_P_1 had developed 41.0, 40.0, and 40.0 nodes, respectively, significantly fewer than the other treatments (Figure 8B). Scion cross-sectional area was similar among treatments for the first 52 DABB (Figure 8C). From 70 DABB, M_P_2 always had the greatest SCA (shoot cross-sectional area), reaching 3.09 cm^2^ at 151 DABB, while M_H_2 had the lowest value of 1.98 cm^2^ at the same date (Figure 8C). The SCA of all other treatments were intermediate from 70 DABB onward (Figure 8C). For the leaf and primary axis development and their dry matter accumulation, H_H_1, H_H_2, M_H_1, and M_H_2 had greater leaf area, almost double that of H_P_1 and H_P_2 which were the smallest (Table 4). A clear division between the total leaf area of ‘PremP009’ and ‘Hosui’ was found, with the exception of M_P_2 that had an intermediate leaf area compared to the other combinations (Table 4). A similar pattern was found for average area per leaf, with a clear difference between ‘PremP009’ and ‘Hosui’. Leaf dry mass generally followed leaf area, with the highest leaf dry weight occurring with ‘Hosui’ leaves; the M_P_2 shoots showed the highest leaf dry weight among the ‘PremP009’ shoot combinations. Even though some considerable differences occurred for the total stem dry mass among shoot treatments, these were not statistically different. The total stem dry weight in: H_H_1 reached the highest value of 163.1 g, while the lowest were H_P_2 and M_P_1 with 120.6 and 119.8 g, respectively. M_P_2 was characteristically greater in all primary stem growth analysis traits compared to other ‘PremP009’ shoots (Table 4). Generally, ‘Hosui’ plants had higher number of leaves though the leaf number of M_P_2 stems was more similar to ‘Hosui’ stems both numerically and statistically. The dry mass per leaf area ratio clearly divided into two groups according to ‘PremP009’ and ‘Hosui’ genotypes, irrespective of tree composition or shoot position. No differences have been found for small spur leaves, stems, and the total small spur dry matter. Finally, in Table 5, the analysis of the remaining tree components below the stem axes (roots, rootstock shank, interstem, scion and budwood axis; refer to Figure 2) did not show any statistical differences.

**Table 4:** Summary of the final dry weight of component parts of primary axes of bi-axis pear trees, considering the interaction between plant type (Homogeneous, H, Mixed, M), genotype (‘Hosui,’ _H_, ‘PremP009,’ _P_) and shoot position (1, lower primary axis; 2, upper primary axis).

	**Primary shoot**					**Small spur^b^**				**Total**		
**Treatment**	**Leaf dry weight (g)**		**Total leaf area (m**^2^**)**		**Average leaf area (cm**^2^**)**		**Stem dry weight (g)**	**Total dry weight (g)**		**Leaves (g)**	**Stem (g)**	**Total (g)**	**Dry weight (g)**	**Leaf number**		**Dry weight/leaf area (g cm**^−2^**)**	
H_P_1	44.5	bc	0.28	c	0.63	b	79.5	124.0	ab	3.2	0.0	3.2	341.1	43.8	b	0.12	b
H_P_2	42.0	c	0.27	c	0.63	b	78.7	120.6	b	1.1	0.0	1.1	350.1	42.8	b	0.13	ab
M_P_1	42.0	c	0.26	c	0.63	b	77.8	119.8	b	0.6	0.0	0.6	381.6	41.7	b	0.14	a
M_P_2	45.9	bc	0.33	b	0.66	b	109.7	155.6	ab	2.5	0.0	2.5	412.4	49.0	a	0.13	ab
M_H_1	56.3	a	0.43	a	0.81	a	97.4	153.7	ab	0.2	0.0	0.2	401.8	52.3	a	0.09	c
M_H_2	53.2	ab	0.40	a	0.78	a	85.4	138.6	ab	0.0	0.0	0.0	395.1	50.7	a	0.10	c
H_H_1	60.3	a	0.42	a	0.81	a	102.8	163.1	a	3.3	3.0	6.3	428.3	51.8	a	0.10	c
H_H_2	57.4	a	0.41	a	0.80	a	97.8	155.3	ab	7.6	8.3	15.9	428.1	51.3	a	0.10	c
Significance	^**^		^**^		^**^		ns	^**^		ns	ns	ns	ns	^**^		^**^	

^a^Mean separation within columns by the Student-Newman-Keuls test; ^*^P < 0.05;

** *P < 0.001; ns: not significant*.

b *no small spurs on H_P_, M_P_ and M_H_ trees*.

**Table 5:** Summary of the final dry weight of roots, rootstock shank, interstem, scion, and budwood parts of bi-axis pear trees, considering the interaction between plant type (Homogeneous, H, Mixed, M), genotype (‘Hosui,’ _H_, ‘PremP009,’ _P_) and shoot position (1, lower primary axis; 2, upper primary axis).

**Treatment**	**Root (g)**	**Rootstock shank (g)**	**Interstem (g)**	**Scion (g)**	**Budwood (g)**
H_P_1	35.8	75.5^1^	30.6^1^	49.1^1^	22.9
H_P_2	48.8	75.5^1^	30.6^1^	49.1^1^	24.5
M_P_1	55.4	93.0^2^	45.7^2^	43.9^2^	23.3
M_P_2	52.4	90.6^3^	51.9^3^	35.8^3^	23.6
M_H_1	51.8	90.6^3^	51.9^3^	35.8^3^	17.8
M_H_2	49.7	93.0^2^	45.7^2^	43.9^2^	24.3
H_H_1	57.5	90.2^4^	45.4^4^	41.5^4^	24.2
H_H_2	54.2	90.2^4^	45.4^4^	41.5^4^	25.6
Significance	ns	ns	ns	ns	ns

*^a^Mean separation within columns by the Student-Newman-Keuls test; ns, non-significant*.

1,2,3,4 *equal number because sharing the same part of the plant*.

## Discussion

Light and carbon response curves (Figures 3A,B) demonstrated differences in leaf photosynthetic response and light efficiency of the plant genotypes ‘PremP009’ and ‘Hosui.’ The experimental unit used (two scion genotypes grafted on the same stock) allowed the comparison of genetic differences in leaf photosyntetic performance. ‘PremP009’ leaves had provided a consistent photosynthetic advantage. The higher efficiency of ‘PremP009’ leaves is represented by the steeper slope of the linear portion of the light response curve, expressing a greater quantum efficiency (Campbell et al., 1992) and a higher maximum rate asymptote. Within the A/C_i_ response, the maximum electron transport rate parameter J_max_ was found to be the key trait increased in ‘PremP009’ leaves compared with ‘Hosui’ (Table 1). In both cultivars, the net assimilation response to internal CO_2_ concentration declined above 1,200 ppm CO_2_, suggesting that at this concentration, a triose phosphate utilization limitation occurred (Sharkey et al., 1986). Differences between the genotypes were also from the maximum CO_2_ assimilation rate of ~36 μmol m^−2^s^−1^ for ‘PremP009’ compared to ~29 μmol m^−2^s^−1^ for ‘Hosui’ (Figure 3B). According to Farquhar and von Caemmerer (1982), the model of CO_2_ assimilation is limited by (i) RuBiSco capacity, (ii) electron transport rate to supply NADPH and ATP for RuBiSco regeneration, (iii) capacity of triose phosphate utilization in starch and sucrose synthesis to regenerate Pi for photophosphorylation. In this case, it seems that in the plateau region (where the curve is constant) photosynthesis is mainly limited by the slow rate of triose phosphate utilization for the formation of sucrose. The limitation in regeneration of RuBP and triose phosphate use provoke the slight decline in transporting electrons out of PSII. This can be the cause of the different shapes of the two curves between leaf genotypes, with a value of ~36 μmol m^−2^s^−1^ for ‘PremP009’ and ~29 μmol m^−2^s^−1^ for ‘Hosui’ (Figure 3B). Moreover, the differences in J_max_ may be elucidated further by considering the differences in leaf mass area (Table 2) of ‘PremP009’ and ‘Hosui’ leaves, because the capacity of carbon assimilation is correlated to LMA (Marini and Barden, 1981). Leaf dry weight and LMA were greater for mature ‘PremP009’ leaves than for mature ‘Hosui’ leaves (Table 2), and this could partly explain the higher J_max_ found because it expresses the maximum rate of electron transport. Further research in sugar metabolism could allow better understanding of this different behavior. The photosynthetic performance differences appear due to RuBP regeneration and triose phosphate utilization, which limited A more in ‘Hosui’ than in ‘PremP009’. Therefore, the “quality” of the electron transport out of PSII determined the rate of photosynthesis of these two pear cultivars. A higher rate of photosynthesis in ‘PremP009’ leaves may send a positive feedback through the electron transport out of PSII, while the lower rate of photosynthesis found in ‘Hosui’ induced inhibitory feedback, which slightly decreased the electron transport. The gas exchange measurements demonstrate that it is possible to use the individual leaf performance of net photosynthesis, stomata conductance and transpiration to elucidate which variables of the grafted plant model system (plant type, genotype and shoot position) influence the photosynthetic performance. In January, differences in photosynthesis depended solely on the leaf genotype influence (Figure 4) because plants were not water stressed, having midday stem water potentials (Figure 4D) equivalent to well-watered young pear trees (Morandi et al., 2014a). In February, besides leaf genotype (Figures 5E,F), leaves of M plants were more active than leaves of H plants (Figures 5G,H), in particular M_P_2 had the highest values (Table 3). Thus, photosynthetic performance of ‘PremP009’ may be affected by the presence of the other scion genotype and the best combination may result from grafting ‘Hosui’ in position 1, closer to the root system as also suggested by dry matter accumulation. It may be possible to assert that ‘PremP009’ leaves could be efficient under strong periods of illumination or high temperature and this behavior could be inherited from the European character by the genetic Asian cross. Possibly, ‘PremP009’ expresses its higher photosynthetic potential most obviously when subjected to extended periods of high irradiance.

The mean total vegetative development of the three plant types at the end of the season showed that H_P_ were less vigorous, gaining less dry matter in the primary shoots than both H_H_ and M possibly because the earlier shoot termination ended node neoformation. In contrast, H_H_ scions continued growing later in the season, developing more nodes, leaves and longer shoots and a greater final tree biomass (Figures 6A,B). M yielded intermediate results, most probably because it is the combination treatment that includes both ‘PremP009’ and ‘Hosui’ genotypes in the one plant.

The ranges of leaf area of ‘PremP009’ in each combination were of 0.26–0.33 m^2^ (Table 4). According to Wünsche et al. (1996), the proportion of intercepted photosynthetic energy converted into biomass relates to photosynthesis and leaf area. Thus, the development of more nodes and greater mean leaf size from early in the season may have increased intercepted solar radiation and dry matter accumulation in trees containing ‘Hosui’ scions (H_H_ and M) compared to homogeneous ‘PremP009’ trees. Considering young newly-grafted apple trees, tree size (dry mass) at the end of the first growing season was found to be strongly dependent on the rate that leaf area increased early in the season (van Hooijdonk et al., 2015). The behavior of H_P_ trees showed slower leaf area formation early in the season, and earlier time of shoot termination, developing shorter shoots, meaning H_P_ shoots had a shorter growth duration. Moreover, ‘Hosui’ trees developed more small sprouts containing additional leaf area. Even if there were no differences in small spur production (Table 4), they could have contributed to light capture necessary for leaf photosynthesis and dry matter accumulation.

‘PremP009’ and ‘Hosui,’ when they are combined with their homologous scion (“H_P_ or H_H_” combination), represent the extreme values in terms of growth and dry matter accumulation. Otherwise, when ‘PREMP009’ is linked to ‘Hosui’ or vice versa (“M” combination), the plant shows average values, revealing how the intermediate treatment is characterized by node number, length and dry matter accumulation placed in between that of homogenous plants (Figures 6A,B). Generally, the proximity of the scion to the rootstock could be an advantage in receiving nutrients and water from roots. In this experiment, M_P_2 primary shoots were 192 cm long with 49 nodes at growth cessation, statistically greater than M_P_1 primary shoots, which were of 157 cm with 42 nodes (Figures 8A,B). Moreover, M_P_2 is the scion combination reaching similar values in length and node number and leaf area to the ‘Hosui’ scion treatments. Considering the dry matter accumulation of H_H_, H_P_, and M plant type (Figure 7), the primary stem dry weight of M_H_1_P_2 is 1.26 times higher than M_P_1_H_2, reaching similar results to the H_H_ plant type. Thus, the growth of ‘PremP009’ stem in M_H_1_P_2, may have been affected by the presence of ‘Hosui’ in the basal location, placed favorably closer to the root system, leading the ‘PremP009’ axis to partition more dry matter to primary shoot leaves and stems, compared with the other ‘PremP009’ shoots. Finally, with an average of 3.09 cm^2^, M_P_2 has the greatest SCA (Figure 8C). Strongly apical dominant pear, as in the case of ‘PremP009’ allocated in position 2, exhibit greater secondary growth (expansion) rather than primary (shoot elongation) and it confirmed that, as asserted by Jackson (2003), distal shoots furthest from the roots exert positional (and usually also a premigenic) dominance over more proximal shoots. Probably the additional growth was due to the presence of ‘Hosui,’ closer to the root system, providing additional carbon for growth.

The experimental bi-axis grafting technique showed a strategic system for a comparative study of ‘PremP009’ photosynthetic performance and related growth analysis: it allowed the comparison between two different leaf-colored pear genotypes bypassing the possible confounding behaviors and alterations due to the plant interaction with environmental factors. Thus, it is possible to determine ‘PremP009’ leaves have a higher photosynthetic advantage, performing a more efficient use of the light intercepted achieved by a more effective RuBP regeneration. ‘PremP009’ leaves are characterized by greater thickness, which may allude to the presence of photosynthetic apparatus and pigments involved in its photochemistry. However, in order to better investigate the photosynthetic capacity of red pigment in pear plants supporting the leaf chlorophylls through unloading energy in excess in the photosynthetic system, additional experiments and analysis are required.

Considering merely the individual leaf photosynthetic yield, ‘PremP009’ leaves showed a higher activity compared to ‘Hosui’ genotype. However, at the end of the season, this is not translated into its final biomass accumulation, because of the effects of seasonal patterns of leaf and node neoformation, which are elementary components of the whole canopy light interception and tree dry matter accumulation. ‘PremP009’ genotype during the first year of growth was characterized by lower vigor with fewer neoformed nodes resulting from earlier shoot growth cessation. This vegetative behavior is likely due to the lower early season leaf area development (and probably a faster leaf senescence) as essential requirements for light interception by young trees. In cropping systems, a low vigor fruit tree genotype is not an undesirable physiological trait; reaching tree maturity earlier in the life of the orchard and with a smaller tree can allow easier management of the orchard. A limitation of this single year experiment is that no information on the leaf genotype photosynthesis x fruit growth interactions were measurable; this knowledge is important considering ‘PremP009’ is likely to be commercially grown in different environmental conditions and managing systems internationally. Unexpected results combining heterogeneous varieties on the same plant came from the interaction between the two genotypes: in the M plants, ‘Hosui’ could be interpreted as having a positive effect supporting ‘PremP009’ growth depending on their respective axis positions in the compound plant. An unverified explanation could be ‘Hosui’ behaves as a mediator and through some hormonal canopy-roots-canopy messaging to stimulate ‘PremP009’ growth, or is simply a feature of additional carbon availability for growth. This mediation action is more noticeable when ‘Hosui’ is grafted in position 1 (closest to the roots system). It would be worth repeating this experiment as a multi-year study to demonstrate if this interaction is reproducible also in field trees. A deeper investigation, following the experimental design of this report, through the evaluation of hormonal messaging, might enable the development of a model for understanding better: (i) hormonal messages for comparing photosynthesis of different scion genotypes; or (ii) hormonal message modulated by the grafting system.

This experiment clearly underlines the strong chain connecting tree physiology and field management studies. A deep understanding on the relationships between these two sciences is needed for creating new options for the interaction of basic and field research and have a clearer and smarter overview of the future horticultural crop horizons.

## Author contributions

All the authors contributed in the drafting of the manuscript and peer review. FT, BvH, DT collected all the field data. LM, FT, BvH, DT designed the experiment and carried out the statistical analysis.

### Conflict of interest statement

The authors declare that the research was conducted in the absence of any commercial or financial relationships that could be construed as a potential conflict of interest.

## Supplementary material

Figure S1 Mean (± Standard Error, SE) midday stem water potential (SWP) considering the plant type (Homogeneous ‘Hosui’ (HH), Homogeneous ‘PremP009’ (HP) and Mixed (M)). Each bar represents the mean ± SE of 12 replicates of the three plant types. Treatments were separated with a one-way ANOVA and compared by the Student-Newman-Keuls test.

Figure S2 Relationship between final tree dry weight and total leaf area of ‘PremP009’ and ‘Hosui’ homogenous plant (H_P_ and H_H_) and mixed plant (M). Each point represents an entire tree.

## Abbreviations

A: net photosynthesis
g_s_: stomatal conductance
E: transpiration
SWP: stem water potential
DABB: day after bud break
RuBisCo: ribulose-1,5-bisphosphate carboxylase/oxygenase
PAR: photosynthetic active radiation
CP: compensation point
SCA: shoot cross-sectional area
LMA: leaf mass area
LDDW: leaf disc dry weight.
