# Supplementary figures and images for: Photosynthetic Performance and Vegetative Growth in a New Red Leaf Pear: Comparison of Scion Genotypes Using a Complex, Grafted-Plant System
Source: Front Plant Sci. 2018 Apr 5;9:404. doi: 10.3389/fpls.2018.00404 (PMC5895778; doi:10.3389/fpls.2018.00404)

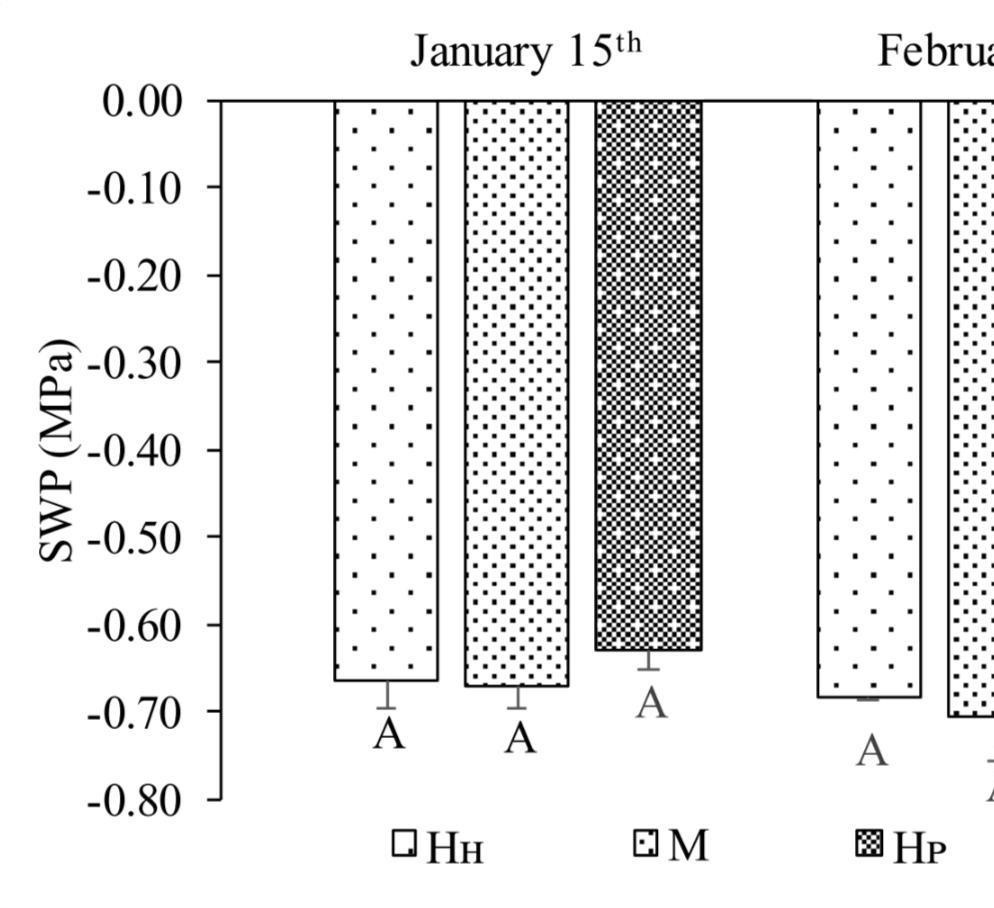

Supplement: Figure S1 — Mean (± Standard Error, SE) midday stem water potential (SWP) considering the plant type (Homogeneous ‘Hosui’ (HH), Homogeneous ‘PremP009’ (HP) and Mixed (M)). Each bar represents the mean ± SE of 12 replicates of the three plant types. Treatments were separated with a one-way ANOVA and compared by the Student-Newman-Keuls test. [file Image1.TIFF]

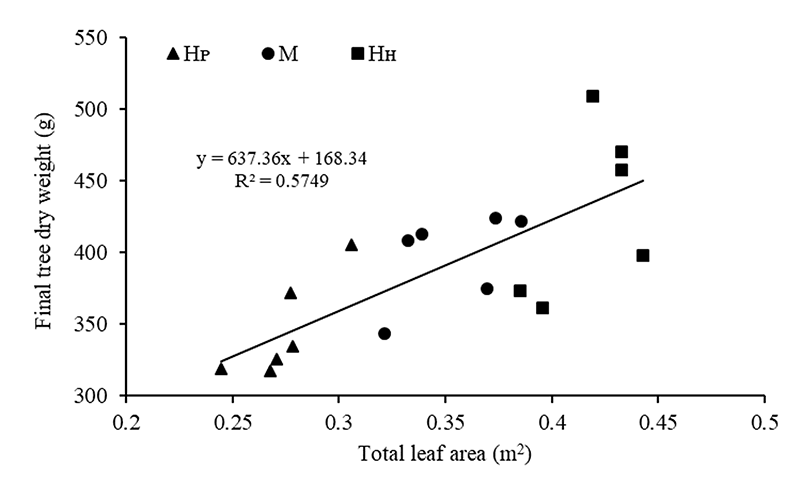

Supplement: Figure S2 — Relationship between final tree dry weight and total leaf area of ‘PremP009’ and ‘Hosui’ homogenous plant (HP and HH) and mixed plant (M). Each point represents an entire tree. [file Image2.TIF]
